# The Stiff Joint: Comparative Evaluation of Monotherapy and Combination Therapy With Urate Lowering Agents in Managing Acute Gout

**DOI:** 10.7759/cureus.45087

**Published:** 2023-09-12

**Authors:** Okelue E Okobi, Helen Oletu, Adaeze B Chukwuedozie-Echeazu, Valentine C Keke, Onyinyechukwu B Nwachukwu, Henrietta S Akunne, Chinwendum U Ekpemiro, Uchechukwu S Oranika, Ngozi T Akueme, Oyewole E Akanle, Buchi C Ogbuagu, Linda A Mbah

**Affiliations:** 1 Family Medicine, Larkin Community Hospital Palm Springs Campus, Miami, USA; 2 Family Medicine, Medficient Health Systems, Laurel, Maryland, USA; 3 Family Medicine, Lakeside Medical Center, Belle Glade, USA; 4 Medicine and Surgery, University of Benin, Benin City, NGA; 5 Public Health, University of Wolverhampton, Wolverhampton, GBR; 6 General Practice, Ebonyi State University, Ebonyi, NGA; 7 General Practice, University of Lagos, Lagos, NGA; 8 Internal Medicine, University of Nigeria, Enugu, NGA; 9 Neurosciences and Psychology, California Institute of Behavioral Neurosciences & Psychology, Fairfield, USA; 10 Family Medicine, American International School of Medicine Georgetown, Guyana, USA; 11 Internal Medicine, Delta State University, Abraka, NGA; 12 Surgery, Federal Medical Centre, Umuahia, NGA; 13 Family Medicine, University of Alberta Hospital, Edmonton, CAN; 14 Dermatology, University of Medical Sciences (UNIMEDTH), Ondo State, NGA; 15 General Practice, Nisa Premier Hospital, Abuja, NGA; 16 Family Medicine, Deer Ridge Family Clinic (DRFC), Calgary, CAN; 17 Primary Care, Veterans Affairs, Fortwayne, USA

**Keywords:** allopurinal monotherapy, lesinurad and allopurinal combination therapy, uricosuric, xanthine oxidase inhibitor, acute gout

## Abstract

Gout, an extremely painful form of arthritis, is triggered by the innate immune system's response to the accumulation of monosodium urate crystals in specific joints and surrounding tissues. This condition is characterized by recurring episodes of excruciating arthritis flares, interspersed with periods of disease quiescence. Over time, gout can result in disability, tophi formation, and severe pain.

The treatment of gout is centered around two main objectives: alleviating inflammation and pain during acute gout attacks and long-term management to reduce serum urate levels and mitigate the risk of future attacks. Addressing inflammation and pain during acute attacks is often complicated by various factors, including underlying health conditions commonly associated with gout, such as hypertension, chronic kidney disease, cardiovascular disease, and diabetes mellitus. Moreover, gout patients are frequently older and have multiple coexisting health issues, necessitating complex medication regimens.

Given the rising prevalence of gout and its associated comorbidities, there's a growing demand for improved treatment options. While existing treatments effectively manage gout in some patients, a significant portion, particularly those with comorbidities, face contraindications to these treatments and require alternative approaches. Innovative medications are required to enhance gout treatment, especially for individuals with concurrent health conditions. These considerations underscore the importance of reviewing both monotherapy and combination therapy approaches for acute gout treatment.

## Introduction and background

Gout attacks are characterized by the rapid onset and intensification of joint pain, often reaching their peak within 24 hours of onset. While these attacks run an acute or chronic course, they tend to start subsiding within 5-12 days without intervention; complete resolution may take longer in certain cases [1,2]. A recent study found that at the 24-hour mark from the onset of an acute gout attack, 16% of participants under placebo experienced over 50% pain reduction, in contrast to the 70% who did not show significant improvement [3]. While most acute gout attacks involve a single joint, a substantial proportion (10%-40%) affect multiple joints [4]. The initial site of involvement in approximately half of all acute gout cases is the metatarsophalangeal joint, a pattern commonly observed among gout patients [4]. Other commonly affected joints during acute gout attacks include the ankle, elbow, wrist, midfoot, fingers, and knee. It's notable that a significant percentage of acute gout patients experience peak pain levels within the first 24 hours, followed by a gradual remission of pain over a period of up to two weeks. Acute and severe gout pain is more frequently observed in individuals with tophi, a longstanding disease, and polyarticular gout.

Gout stands as one of the most prevalent forms of inflammatory arthritis affecting adults in the United States [5]. Notably, about 10% of individuals aged 65 and above report experiencing gout, in contrast to the overall gout prevalence rate of nearly 4% in the US population [1]. Similarly, in the United Kingdom, the incidence rate of gout is estimated at 2.68 per 1000 person-years, with a noticeable increase in prevalence with advancing age [6]. Beyond its high frequency, acute gout's impact extends to diminished quality of life and heightened utilization of healthcare services. Inadequate management of acute gout can result in recurrent hospital admissions and eventual disability [7]. However, effective gout management often encounters challenges driven by various barriers such as misconceptions held by physicians and patients as well as the requirement for familiarity with evidence-based treatment, management guidelines, and practices [8,9].

The onset of gout is underpinned by a biochemical abnormality known as hyperuricemia, denoting a serum urate (SU) concentration equal to or surpassing 6.8 mg/dl (0.408 mmol/l). Typically, hyperuricemia arises from an excess of urate production or insufficient urate excretion, accounting for approximately 90% of gout patients' hyperuricemia cases [10]. The risk of acute gout development is closely associated with the severity of hyperuricemia. However, it's important to note that hyperuricemia alone is not sufficient to trigger gout, as demonstrated by several US-based studies indicating a hyperuricemia prevalence of 21% alongside a gout prevalence of 3.9% [5]. Given the established connection between gout development, hyperuricemia, and the efficacy of serum urate reduction in gout management, urate-lowering therapy (ULT) stands as a fundamental cornerstone in the treatment of acute gout. The inadequacy of existing ULT agents has led to persistent unmet needs for effectively addressing hyperuricemia in gout. Additionally, the emerging evidence linking hyperuricemia with metabolic and cardiovascular comorbidities has spurred a growing interest in the development of novel and potent ULTs [11,12]. 

In the context of reducing urate levels, the primary approach entails employing febuxostat or allopurinol, both of which are categorized as xanthine oxidase inhibitors responsible for curtailing urate production. Nonetheless, despite these treatment options being put forth, achieving the desired urate blood levels has remained challenging for many patients. Shedding light on this, a recent clinical trial encompassing 324 individuals experiencing acute gout subjected to both monotherapy and combination therapy revealed noteworthy insights.

Lesinurad, as a novel agent in the management of hyperuricemia and gout, has shown promise in enhancing urate level control when used in combination with xanthine oxidase inhibitors. Notably, a larger proportion of patients undergoing combination therapy, specifically involving lesinurad in conjunction with a xanthine oxidase inhibitor, successfully reached the targeted urate levels compared to those on a monotherapy regimen of febuxostat [13]. This study also elucidated that lesinurad functions by impeding the uric acid transporter in the kidneys while augmenting uric acid excretion through urine [13]. Through this combined approach, utilizing both lesinurad and XOI, a dual strategy emerges for effectively controlling and diminishing blood urate levels.

Addressing combination therapy, Clarson et al. assert that the root cause of gout lies in the accumulation of urate crystals within joints, giving rise to inflammation and pain [14]. In certain gout patients, this process escalates, leading to the formation of crystal tophi deposits that exacerbate joint damage and inflammation. To address this scenario, guidelines have recommended combination therapy involving a xanthine oxidase inhibitor (XOI) alongside a uricosuric agent for patients with acute gout who cannot achieve management and treatment targets through XOI monotherapy alone. Notably, existing combination treatment guidelines advocate for tophaceous gout patients to attain urate blood levels below 5 mg/dl to mitigate potential adverse events [14-17]. This study's primary objective is to evaluate the efficacy of urate-lowering agents, both in singular and combined therapy, for the management of acute gout. It seeks to compare the impact of these treatment strategies on key parameters such as serum urate level reduction, relief from gout symptoms, including pain and tophi, and overall disease management. Emphasizing the necessity for enhanced treatment options, particularly for individuals with concurrent health issues, the study examines the roles of xanthine oxidase inhibitors (allopurinol, febuxostat) and uricosuric agents (lesinurad) in optimizing outcomes for acute gout patients. As the debate between monotherapy and combination therapy involving urate-lowering agents continues to evolve, this systematic review fills a crucial gap in understanding.

## Review

Methods and materials

This systematic review employed a comprehensive literature review approach. Major global databases, including Web of Science, EMBASE, PubMed, Cochrane Library, HINARI, and Google Scholar, were systematically searched. Additionally, reference lists of previously identified literature were examined to gather more relevant studies. The Preferred Reporting Items for Systematic Reviews and Meta-Analyses (PRISMA) guidelines were followed for methodological rigor. A thorough search was conducted using Boolean operators and various keywords such as allopurinol monotherapy, lesinurad and allopurinol combination therapy, uricosurics, xanthine oxidase inhibitors, and acute gout. Inclusion criteria encompassed studies published between 2000 and 2023.

Eligibility criteria

Studies published up to 2023 were included. The eligibility criteria consisted of the following: study participants were adults; observational study designs, including case-control and cross-sectional studies, were considered; and human studies conducted in regions with Jehovah Witness groups were included.

Data extraction

Data were extracted from diverse studies and full-text articles by the author. Discrepancies were resolved through discussions and expert consultations. Key information, such as the main author, publication year, study location, sample size, response rate, and screening tools employed, was independently collected in a standard format.

Quality assessment

The quality of the included studies was evaluated using the Joanna Briggs Institute quality assessment tool. Studies were scored based on predefined criteria using responses like yes, no, unclear, and not applicable. Each study's overall quality score was calculated based on the total positive scores.

Statistical analysis

Comprehensive systematic review software version 3 was utilized for statistical analyses. Prevalence rates from individual studies were pooled using a random effects systematic review. Heterogeneity between studies was assessed with I2 statistics, where values of 25%, 50%, and 75% indicated low, medium, and high heterogeneity, respectively. Attributes like sample size, research design, publication year, and study location were analyzed to identify potential sources of heterogeneity across studies.

Results

**Figure 1:**
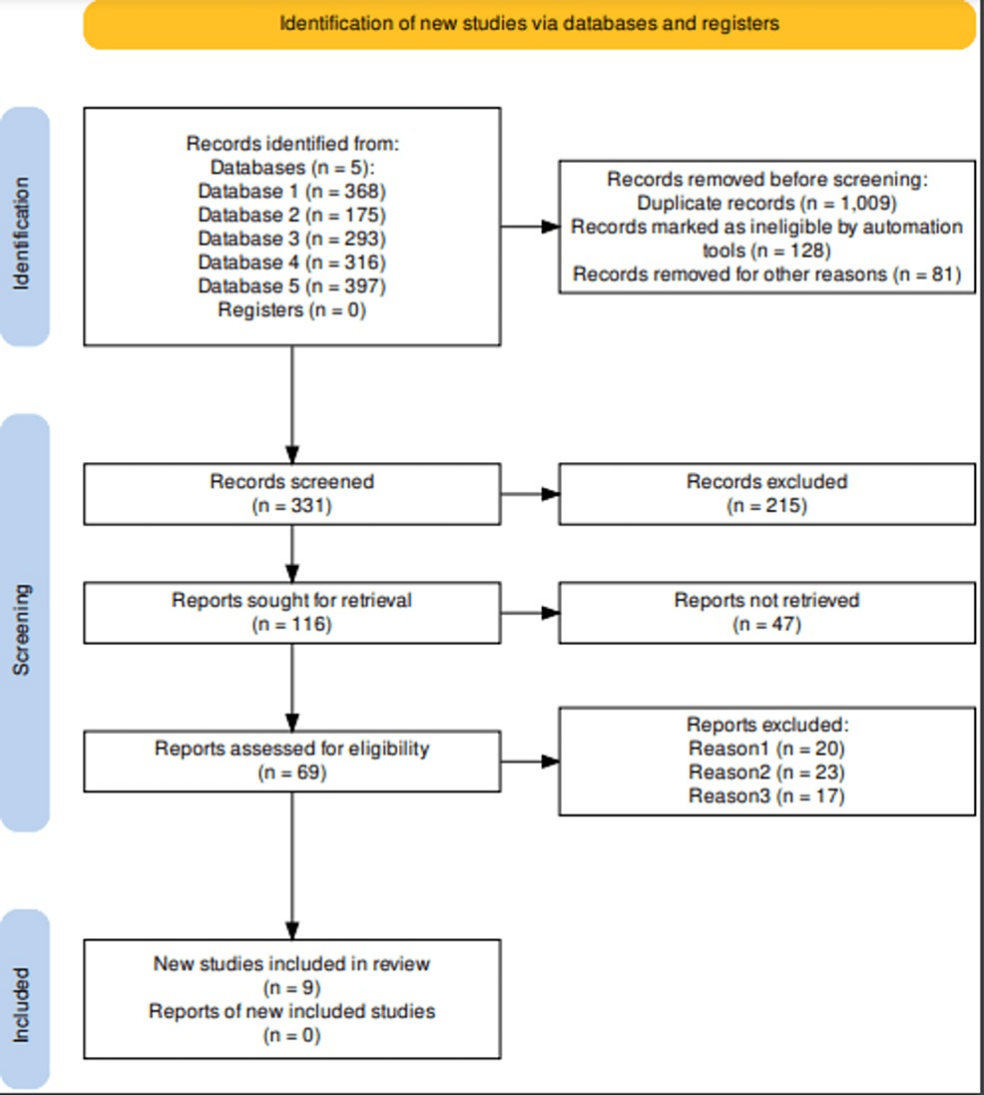
PRISMA flow diagram for the systematic review PRISMA flow diagram indicating the study selection process for the present systematic review.

The thorough search across the identified electronic databases yielded 1549 records. Among these, 1009 duplicates were removed, and 128 were excluded due to ineligibility. An additional 81 records were excluded for various reasons, including redundancy. The abstracts and titles of 331 articles were assessed, leading to the exclusion of 215 articles. A total of 116 articles were retrieved for full-text assessment, resulting in the exclusion of 47 non-retrievable articles. Ultimately, 69 articles underwent full-text screening, and only nine met the criteria for inclusion in this systematic review, as illustrated in the PRISMA flow diagram in Figure 1. 

The assessment of the effectiveness of both monotherapy and combination therapy was centered around observing changes in serum urate (sUA) levels, tophi presence, gout flare occurrence rates, and urinary uric acid levels. Safety evaluations encompassed monitoring adverse events and conducting laboratory assessments. Starting from initial studies in the 1960s characterized by open-label designs and case reports to more recent and meticulously structured studies with larger patient populations, consistent outcomes have emerged. These findings consistently highlight that combination therapies involving both a xanthine oxidase inhibitor (XOI) and uricosuric agents yield significantly greater reductions in serum urate levels compared to the urate-lowering effects achievable through monotherapy in acute gout treatment. This heightened reduction in serum urate levels corresponds to improved control over acute gout symptoms, including a more enhanced resolution of tophi. Table 1 lists down all the studies reviewed in the systematic review. 

**Table 1:** List of the selected studies reviewed in this systematic review. The selected studies for the systematic review include the author name, study title, study objective, sample size, year of publication, and publishing journal.

Author/s	Study title	Objective	Design	Sample	Publication year	Journal
Fan et al. [17]	Comparison of efficacy and safety of urate-lowering therapies for hyperuricemic patients with gout: a meta-analysis of randomized, controlled trials.	To evaluate the efficacy and safety of the commonly used urate-lowering therapies (ULTs): febuxostat, allopurinol, and lesinurad in hyperuricemic patients with gout.	Meta-Analysis	7968 patients	2021	Clin Rheumatol.
Bardin et al. [18]	THU0537 clinical response of tophus and flares to extended use of lesinurad in combination with a xanthine oxidase inhibitor in patients with gout.	To evaluate the impact of long-term treatment with lesinurad and XOI on tophus and flares for at least one year and up to two years.	Mixed method research	471 patients	2016	Arthritis Rheumatol.
Saag et al. [19]	Lesinurad combined with allopurinol: a randomized, double‐blind, placebo‐controlled study in gout patients with an inadequate response to standard‐of‐care allopurinol (a US‐based study).	To evaluate the effectiveness of daily lesinurad (200 mg or 400 mg) and allopurinol versus placebo plus allopurinol in reducing urate levels in patients with serum urate (UA) levels above a target of <6.0 mg/dl.	Mixed method research	603 patients	2017	Arthritis & rheumatology,
Dalbeth et al. [20]	Efficacy and safety in patients with tophaceous gout receiving lesinurad and febuxostat combination therapy: interim analysis of an extension study.	To assess the efficacy and safety of lesinurad combination therapy in patients for up to two years.	RCT	235 patients	2015	Arthritis & Rheumatology
Tausche et al. [21]	Lesinurad monotherapy in gout patients’ intolerance to a xanthine oxidase inhibitor: a six-month phase 3 clinical trial and extension study.	To investigate the efficacy and safety of lesinurad, a selective uric acid reabsorption inhibitor, in a six-month, phase 3 clinical trial and extension study.	RCT	214 patients	2017	Rheumatology
Becker et al. [22]	The urate-lowering efficacy and safety of febuxostat in the treatment of the hyperuricemia of gout: the CONFIRMS trial.	To compare the urate-lowering efficacy and safety of febuxostat and allopurinol in subjects with gout and serum urate (sUA) > or = 8.0 mg/dL in a six-month trial.	RCT	2269 patients	2010	Arthritis research & therapy
Pruis et al. [23]	Cost-effectiveness of sequential urate-lowering therapies for the management of gout in Singapore.	Conduct a cost-utility analysis of sequential ULT treatment strategies for gout, including strategies with and without HLA-B*58:01 genotyping, before treatment initiation, with a view to determining optimal gout management.	Systematic review and meta-analysis	12 therapies for gout management	2020	Journal of Medical Economics
Alghamdi et al. [24]	An overview of the role of xanthine oxidase inhibitors in gout management.	To discuss the role of XO inhibitors in treating gout disease and provide a review of the different available XO inhibitor medications.	Literature review	N/A	2021	Arch. Pharm. Pract.
Hu and Brown [25]	Comparative effect of allopurinol and febuxostat on long-term renal outcomes in patients with hyperuricemia and chronic kidney disease: a systematic review.	To assess the long-term renal outcomes of allopurinol compared with febuxostat in patients with hyperuricemia, and CKD, or kidney transplantation.	Systematic review	Three retrospective observational studies	2020	Clinical Rheumatology

Discussion

In acute gout, the desired clinical outcomes entail the prevention and eventual eradication of acute gouty arthritis, the dissolution of tophi, and the prevention of severe arthropathy. The European League Against Rheumatism (EULAR) and the American College of Rheumatology (ACR) 2012 guidelines, alongside the 3-e rheumatology recommendations, have been endorsed as an effective treat-to-target strategy to facilitate the realization of positive clinical outcomes [1,15,16]. The approved and widely accepted target level of serum urate is <6 mg/dL, even though a much lower target level of serum urate of <5 mg/dL is mainly preferred for individuals with acute gout requiring faster serum urate crystal dissolution, marked by the existence of severe arthropathy or tophi, and for those patients with high-frequency gout attacks. On the contrary, the American College of Physicians (ACP) clinical practice guidelines for 2017 regarding acute gout treatment and management have not endorsed any target serum urate level. ACP guidelines maintain that the evidence is inadequate to conclude if the advantages of the treat-to-target strategy dwarf the various harms linked to medication escalation and continual serum urate level monitoring. However, ACP guidelines have recommended the “treat-to-avoid symptoms" strategy, which aims to avoid recurrent acute gout attacks without the need to monitor serum urate levels [2].

As noted earlier, the therapies for acute gout aim to reduce pain while promoting early and complete resolution. The ACR has recommended pharmacological therapy as the appropriate first-line treatment, along with adjunctive topical ice and plenty of rest [2]. Characteristically, monotherapy is considered appropriate when the patient experiences either mild or moderate pain that affects not more than two joints. On the other hand, chronic pain and attacks that affect several joints simultaneously are highly likely to benefit from combination therapies as opposed to monotherapy. Therefore, in reviewing the effectiveness of monotherapy in the management and treatment of acute gout, Fan et al. ascertain that clinical guidelines emphasizing XOI monotherapy, with either febuxostat or allopurinol, should be considered as the initial-line therapy for critical gout prevention [17]. Even though such guidelines have not proposed any XOI over others, allopurinol has been widely prescribed for acute gout, attributed to the drug's cost-effectiveness and generic availability [18]. Nevertheless, studies comparing allopurinol and febuxostat have disclosed significantly higher serum urate attainments with febuxostat [19,20]. However, it is essential to note that such data are limited by the suboptimal allopurinol dosing secondary to physicians' dearth of additional dose escalations. Further, Ruoff and Edwards in their study indicated that when an allopurinol dose of 300mg/day was utilized in a head-to-head comparison study against a real-life average allopurinol dosage of 184.9mg/day, the serum urate target achievement was below 50% with the usual dosage in the controlled trial [1]. The outcomes of the study were undesirable. The success rates of allopurinol as a monotherapy drug noticeably improved when the dosage was escalated to dosages that were higher than 300mg/day [21,22].

As a monotherapy treatment, the allopurinol conservative dosing approach, which entails the use of less than 300mg/day, is a result of aspects that include the product labeling dosage limitations related to renal impairments as well as concerns of potential adverse side effects, including allopurinol hypersensitivity syndrome [AHS], which, despite being infrequent, is fatal. Even though it is vital to consider such safety concerns before allopurinol initiation, the escalation of the dosage to over 300mg/day has been reported to be safe [22,23]. However, to minimize the potential risk of AHS, the initial doses of allopurinol as monotherapy should not be over 100mg/day for any gout patient and 50mg/day for acute gout patients with either stage 4 or, worse, chronic kidney disease. As such, progressive titration every 2 to 5 weeks is recommended to ensure that dosages over 300mg/day may be utilized in acute gout patients with renal disease and impairment in cases accompanied by adequate monitoring and education of the patients [24]. Moreover, Ruoff and Edwards maintain that allele HLA-B5801 testing is vital before allopurinol initiation, particularly in patients with either stage 3 or worse chronic kidney disease, and that the use of allopurinol must be avoided in individuals who tested positive for the HLA-B5801 allele, owing to the increased risk of AHS [1].

Consequently, febuxostat does not need any adjustment or escalation of the renal dose, even as its usage has been reported not to have a comparable risk for AHS as allopurinol. Nonetheless, safety trials' preliminary outcomes have indicated an increment in cardiovascular mortality in instances where a febuxostat was compared against allopurinol [24,25]. The trial design was aimed at comparing the cardiovascular mortality rates, non-fatal stroke, non-fatal myocardial infarction, and unstable angina that needs critical coronary revascularization between allopurinol 200 to 600mg/daily and febuxostat 40 and 80mg/daily over a five-year duration [25]. The primary endpoint of composite cardiovascular events was not more significant in the febuxostat group. However, when the outcomes were evaluated individually, febuxostat showed higher rates of cardiovascular-related death and death from all causes. The FDA will determine the final results from the manufacturer when they are available. These data do not suggest that complete avoidance or immediate discontinuation of febuxostat is necessary. Instead, healthcare providers should evaluate patient-specific cardiovascular risk versus the benefits of febuxostat therapy. When not contraindicated, allopurinol may be preferred.

Typically, allopurinol, a xanthine oxidase inhibitor (XOI), is the primary therapy for managing gout, with febuxostat as a second-line option for curbing urate production. However, clinical studies have demonstrated that only around 40% of patients with acute gout receiving allopurinol at a dosage of 300 mg/day achieve the desired serum urate levels (<6.0 mg/dL) [26]. In contrast, febuxostat, administered at dosages ranging from 80 to 240 mg/day, helps approximately 48% to 69% of patients attain the target serum urate level; nonetheless, a substantial portion of patients in these trials struggled to reach and sustain the desired levels [27]. In situations where adequate doses of XOI fail to achieve the intended serum urate levels, the European League Against Rheumatism (EULAR) recommends alternative treatments. These alternatives include switching the XOI, combining the XOI with uricosuric medications, or transitioning to a uricosuric agent [28].Hence, the combination of an XOI and lesinurad serves as a suitable approach, addressing the shortcomings of the aforementioned therapies for gout patients with insufficient responses to XOIs [23,25,27,28]. This combination provides a dual mechanism for lowering serum urate levels: it inhibits renal urate reabsorption and subsequently reduces urate production. Notably, the lesinurad-XOI combination effectively targets URAT1, a uric acid transporter, contributing to increased urate anion reabsorption within renal tubules [29,30]. By obstructing URAT1, the combination enhances the overall rate of urate excretion while simultaneously lowering serum urate concentrations. Furthermore, the combined therapy also hampers OAT4, an organic anion transporter implicated in luminal urate reabsorption, which is linked to diuretic-induced hyperuricemia [29,31-36].

Lesinurad, an emerging uricosuric agent, effectively enhances renal urate excretion by selectively inhibiting the action of renal uric acid transporter 1 (URAT1). Its application is indicated for adult patients, in conjunction with an XOI, to provide supplementary treatment for hyperuricemia associated with gout. This is especially beneficial for those who have not achieved the desired target serum uric acid (UA) levels through an appropriate dose of XOI treatment alone. By adopting this combined approach, the potential to achieve serum UA targets is realized through the inhibition of new crystal formation and the facilitation of the dissolution of existing crystals. As a result, this strategy contributes to improved outcomes, including reduced flares and the resolution of tophi. In light of pharmacodynamic effects, investigations involving healthy individuals have divulged significant insights. In particular, a combination regimen consisting of lesinurad (200mg) and an XOI led to noteworthy reductions in serum urate levels and notable enhancements in renal clearance and fractional excretion [27]. Specifically, when lesinurad was administered at 200mg alone, serum urate levels exhibited an average decrease of approximately 46% and 26% at 6-hours and 24 hours post-dosage, respectively [30,31]. However, when lesinurad (200mg) was administered in combination with an XOI, an additional reduction of 25% and 19% in serum urate levels was observed at 6 and 24 hours post-dosage, respectively [29,31]. These findings were further substantiated by pivotal studies, which are discussed in more detail below.

To assess the comparative effectiveness of outcomes between monotherapy and combination therapy, a randomized, multicenter, double-blind, and placebo-controlled phase III study was conducted over a 12-month period in the United States [19]. The primary objective was to evaluate the impact of lesinurad (200-400mg/day) in combination with allopurinol against a placebo in combination with allopurinol, specifically in patients with elevated serum urate levels who had not sufficiently responded to allopurinol monotherapy [19]. The study population included patients on a minimum dose of 300mg/day allopurinol (200mg/day for those with moderate renal impairments), with serum urate levels exceeding 6.5mg/dL during screening and having experienced two or three gout attacks in the past year [19]. The study findings aligned with previous research [31-34], demonstrating that the combination therapy of lesinurad and allopurinol yielded more pronounced reductions in serum urate levels compared to monotherapy involving allopurinol alone [32-34]. Similarly, the CLEAR 1 study identified the primary endpoint as the proportion of patients achieving the target serum urate level of <6.0 mg/dL within the sixth month [35]. Furthermore, significant secondary endpoints included acute gout attack rates requiring treatment from the 7th to the 12th month, as well as the percentage of patients reporting complete resolution of a target tophus at the 12th month [35].

In their comprehensive study involving a total of 2377 evaluated patients, Saag et al. conducted a double-blind, randomized trial encompassing 607 patients who were administered either 200mg/day or 400mg/day of lesinurad, as well as a placebo group in a 1:1:1 ratio distributed across 138 study locations. An additional 603 patients received a single dose of lesinurad [19]. Over the initial five months, patients received gout attack prophylaxis from the 14th day following screening randomization. The study revealed that among the patients, the proportion achieving the targeted serum urate level of <6.0 mg/dL at the primary endpoint or by the sixth month was 27.9% in the allopurinol monotherapy group, while it was notably higher at 54.2% in the combination therapy group receiving 200mg lesinurad in conjunction with allopurinol [19]. This data pointed to a statistically significant difference at the sixth month or primary endpoint between acute gout patients who underwent combination therapy with 200mg of lesinurad and allopurinol compared to those on allopurinol monotherapy (p<0.0001) [19]. These findings were further substantiated by a 12-month study, wherein monthly assessments indicated that the percentage of patients achieving the target serum urate level of <6.0 mg/dL between the 1st and 12th months was significantly higher in patients undergoing combination therapy with either 200mg or 400mg lesinurad and allopurinol, as opposed to the monotherapy allopurinol group (p<0.0001 for each comparison) [18].

In another recent large-scale study spanning South Africa, Europe, North America, Australia, and New Zealand and involving 2199 acute gout patients, 611 patients were randomly allocated to three groups: 200mg lesinurad, 400mg lesinurad, and placebo, in a 1:1:1 ratio distributed across 152 study locations. An additional 610 patient participants received a single dose of lesinurad medication [20]. The percentage of patients achieving the target serum urate level (<6.0 mg/dL) by the sixth month or primary endpoint was 23.3% in the monotherapy allopurinol-only group and a significant 55.4% in the combination therapy group receiving 200mg lesinurad alongside allopurinol [36]. Notably, statistically significant differences were observed in the combination therapy group compared to the monotherapy group at the sixth month (p<0.0001). Similarly, statistically significant differences (p<0.0001) were noted at the sixth month or primary endpoint when comparing the monotherapy allopurinol with placebo groups to the 400mg lesinurad and allopurinol cohorts (67%) [20,21]. Moreover, from the 1st to the 12th month, the percentage of patients achieving the target serum urate level was consistently higher in the combination therapy groups involving lesinurad and allopurinol, in contrast to the monotherapy allopurinol group, at each monthly assessment (p<0.0001, for every comparison) [21]. Additionally, throughout the entire 12-month treatment duration, the combination therapy groups consistently exhibited lower average serum urate levels compared to the monotherapy allopurinol group (p<0.001 for every comparison) [20].

Subsequently, another pivotal study that delved into the comparative effectiveness of monotherapy versus combination therapy for acute gout treatment was carried out by Dalbeth et al. This 12-month investigation also sought to assess the safety and efficacy of the combined approach involving lesinurad and febuxostat in managing acute gout. Initial trials focusing on the combination therapy of lesinurad and febuxostat had already revealed more substantial reductions in serum urate levels compared to febuxostat monotherapy [24]. Consequently, the study enrolled adult patients experiencing acute gout who had previously been treated with serum urate-reducing agents or were treatment-naive. Eligibility criteria included baseline blood serum urate levels of ≥8.0 mg/dL for patients not on urate-reducing agents and ≥6.0 mg/dL for those actively receiving such therapy [32-36]. Participants were required to have at least one tophus measuring between 5 and 20mm in diameter, located either in the feet or hands. They were subsequently randomized into three groups: febuxostat 80mg and placebo; lesinurad 200mg and febuxostat 80mg, and lesinurad 400mg and febuxostat 80mg; in a 1:1:1 ratio. Among the 1045 initially evaluated, 330 were randomized, and 324 received at least a single dose of the study medication [36]. The analysis revealed that at the sixth month, 46.8% of participants in the febuxostat monotherapy group achieved the target blood serum urate level of <5.0 mg/dL, while the combination therapy group with 200mg lesinurad and febuxostat achieved a higher percentage of 56.6% [36]. However, no statistically significant difference was observed between the combination therapy of 200mg lesinurad and febuxostat and the febuxostat monotherapy (p=0.13). The researchers also conducted a pre-specified analysis, focusing on a subgroup of patients with a baseline blood serum urate level of ≥5.0 mg/dL after three weeks of febuxostat monotherapy (n=161). In this subgroup, 24% of patients achieved the target serum urate level at the sixth month through febuxostat monotherapy, while 44% of those on the combination therapy of 200mg lesinurad and febuxostat reached the target (p=0.024 compared to febuxostat monotherapy) [36]. Additionally, no statistically significant difference was observed in serum urate levels with the combination therapy of 200mg lesinurad and febuxostat at the sixth month, yet significant treatment effects were evident in this subgroup at every other monthly assessment from the 1st to the 12th month (p≤0.0281) [37-39].

Furthermore, the study noted that the percentage of patients reporting complete resolution of the target tophus or tophi was slightly higher in the combination therapy groups: 25.6% in the lesinurad 200mg and febuxostat group and 21.1% in the lesinurad 400mg and febuxostat group. However, this difference was not statistically significant [37]. Remarkably, at the 12th month, the targeted tophi area reductions in the combination therapy groups-lesinurad 200mg and febuxostat, as well as lesinurad 400mg and febuxostat-were reported as 50.1% and 53%, respectively, in comparison to 28.3% in the febuxostat monotherapy group [38-39].

Limitations of this review

The current systematic review highlights several potential limitations, many of which are inherent to the pharmacological study model employed. As there is no direct head-to-head comparison between monotherapy and combination therapy for acute gout treatment, the diverse clinical parameters have been sourced from various studies, all of which are underpinned by published literature with robust clinical evidence and the validation of rheumatology experts. However, the absence of suitable comparative clinical trials has prompted the utilization of indirect comparisons across treatment modalities. While these comparisons provide significant evidence, they also serve as invaluable analytical tools, enhancing our understanding of the effectiveness of monotherapy and combination therapy in the context of acute gout treatment.

Another noteworthy limitation pertains to the lack of assessment regarding potential risks such as toxicity, morbidity, or mortality associated with the studied models, particularly in the case of combination therapies for managing acute gout. Although a recent study has suggested a link between higher serum urate levels and increased mortality risk when compared to the general population, our current study model does not specifically address these risks or their impact. Therefore, while the systematic review offers valuable insights into the comparative effectiveness of different treatment approaches, the broader risks and outcomes associated with these therapies are not fully explored within the scope of this study.

## Conclusions

Several authors have discussed the potential benefits of combination therapy involving a uricosuric and an XOI in achieving greater serum urate reduction compared to monotherapy. However, a consensus on the superiority between combination and monotherapy has yet to be reached. Combination therapy could prove advantageous in attaining therapeutic targets for patients with acute gout who struggle to achieve target serum urate levels through monotherapy or those unable to tolerate XOIs at monotherapy doses. Notably, the combination of lesinurad and allopurinol has demonstrated superiority over febuxostat monotherapy in reducing serum urate levels below 6 or 5 mg/dL. Consequently, this combination therapy offers a dual-action mechanism, effectively inhibiting both renal urate reabsorption and urate production. More well-designed randomized clinical trials may be needed in this regard to explore the potential benefits and demerits thereof. Furthermore, this study offers significant insights into the management of acute gout through a comparative evaluation of treatment approaches. Its findings have practical implications for clinicians and call for continued research to further refine gout treatment strategies.
